# Expression of gremlin1 in gastric cancer and its clinical significance

**DOI:** 10.1007/s12032-017-1073-4

**Published:** 2018-02-02

**Authors:** Yoichi Yamasaki, Sumiya Ishigami, Takaaki Arigami, Yoshiaki Kita, Yasuto Uchikado, Hiroshi Kurahara, Yuko Kijima, Kosei Maemura, Shoji Natsugoe

**Affiliations:** Department of Digestive Surgery, Breast and Thyroid Surgery, Kagoshima University School of Medicine, 8-35-1 Sakuragaoka, Kagoshima, 890-8520 Japan

**Keywords:** Gremlin1, Gastric cancer, Immunohistochemistry, Survival

## Abstract

As an antagonist of bone morphogenetic proteins (BMPs), 2, 4 and 7, gremlin1 plays a role in regulating organogenesis, tissue differentiation and angiogenesis. However, there is little information regarding gremlin1 in gastrointestinal cancer. We attempted to clarify how gremlin1 expression affects the clinical features and biological properties of gastric cancer. A total of 232 gastric cancer patients who received R0 gastrectomy at Kagoshima University Hospital were enrolled. Gremlin1 expression in gastric cancer was detected by immunohistochemical and western blotting methods. Correlations between clinicopathological parameters and gremlin1 expression were analyzed. Gremlin1 was identified in the cytoplasm and nucleus of all gastric cancer cell lines and some regions of surgical specimens of gastric cancer. One hundred and seventeen of the 232 patients (50.4%) were classified as gremlin1-positive based on gremlin1 expression. Gremlin1 positivity was correlated with shallower tumor depth, smaller tumor size, less nodal involvement and vessel invasion (*p* < 0.05). The 5-year survival rate of the gremlin1-positive group was 81%, which was significantly higher than the gremlin1-negative group (*p* < 0.01). Multivariate analysis revealed that gremlin1 was not selected as an independent prognostic marker. Gremlin1 expression in gastric cancer may be a useful prognostic marker that is involved with the BMP signaling pathway. Furthermore, gremlin1 may have clinical use as a diagnostic and treatment tool.

## Introduction

Gastric cancer is the second most common cancer-related cause of death worldwide. East Asian countries, including Japan, are among the most high-risk areas for gastric cancer [1]. Following the development of endoscopic instruments and diagnostic skills, the detection rate of early gastric cancer has increased, and patients are treated with less invasive surgical procedures, such as endoscopic resection or laparoscopic gastrectomy [2]. We now have promising clinical results for gastric cancer in Japan. For patients with gastric cancer who undergo curative resection, postoperative relapse often occurs. Thus, the postoperative outcome of advanced gastric cancer remains poor [3]. The TNM classification consists of tumor depth, nodal and distant metastasis with distant metastasis as the strongest prognostic marker [4]. The classification also includes peritumoral lymphatic and venous invasions, which are linked to aggressive tumor behavior in gastric cancer [5, 6]. Additional biomolecular prognostic markers are currently being investigated by several clinical researchers. Molecules regulating cell adhesion [7–9], cell cycle and the signaling pathway of tumor proliferation [10, 11] are representative prognostic markers for gastric cancer.

We indicated that bone morphogenetic protein-7 (BMP7) is one of the independent prognostic markers in gastric cancer [11]. Based on this result, we attempted to highlight the role of gremlin1. Hsu was the first to report that gremlin1 was an antagonist of bone morphogenetic proteins (BMPs) [12]. Gremlin1 is thought to prevent ligands from interacting with their receptors, which results in the inhibition of transforming growth factor-beta signaling. As an antagonist of BMP proteins, gremlin plays a role in regulating organogenesis, body patterning and tissue differentiation [13, 14]. Gremlin1 works to bind vascular endothelial growth factor receptor-2 (VEGFR2) and modulates tumor angiogenesis and possibly putative angiogenesis-modulating gene [15]. Expression of gremlin1 is observed in multiple normal adult and tumor tissues, such as the skin, stomach, lung, kidney and testis [16]. Recent studies have indicated that gremlin1 was correlated with the biological behavior of some cancer types [17]. However, there have been no reports regarding how gremlin1 expression affects the biological characteristics of gastric cancer. In this study, we investigated gremlin1 expression in gastric cancer and discuss its clinical implications.

## Materials and methods

A total of 232 patients with gastric adenocarcinomas which had invaded deeper than the submucosal layer were enrolled in this study. The patients consisted of 160 males and 72 females, and the mean age was 66 years (from 31 to 85 years). All patients received curative gastrectomy with lymph node dissection at Kagoshima University Hospital between 1996 and 2013. None of the patients had preoperative chemotherapy. The number of patients at the final pathological stage of I, II, III and IV was 69, 45, 63 and 55, respectively. One hundred and eighteen patients were classified as having differentiated adenocarcinoma (papillary, well-differentiated and moderately differentiated tubular adenocarcinoma), and 114 as undifferentiated adenocarcinoma (poorly differentiated adenocarcinoma, mucinous adenocarcinoma and signet-ring cell carcinoma). The study was approved by the Institutional Review Board of Kagoshima University School of Medicine. Written informed consent was obtained from all patients, and the study was approved by our institutional ethics committee. This investigation conformed to the principles outlined in the Declaration of Helsinki. Clinicopathological factors were assessed by the Japanese Classification of Gastric Carcinoma [18].

### Immunohistochemistry for surgical specimens

Paraffin-embedded sections of tumor nests obtained through surgery were sliced at a thickness of 4 μm to facilitate immunohistochemical analysis. After deparaffinization and dehydration, the sections were heated at 121 °C for 10 min for antigen retrieval. Sections were soaked in PBS prior to immunohistochemical analysis. The sections were also soaked in 0.3% H_2_O_2_ for 30 min in order to block endogenous tissue peroxidase, which was followed by treatment with bovine serum for 30 min to reduce nonspecific binding. The gremlin1 antibody (rabbit polyclonal; PAB14845; Abnova) was diluted to 1:200 and left overnight at 4 °C. Sections were rinsed in PBS and visualized by standard techniques for labeled avidin–biotin immunoperoxidase staining. Gremlin1 was subsequently visualized using a DAB Substrate Kit. The slides were briefly counterstained with hematoxylin and mounted aqueously. Well-differentiated adenocarcinoma of the colon was used as a positive control for gremlin1 expression. Gremlin1 expression from five gastric cancer cell lines was also examined immunohistochemically in the same manner without deparaffinization.

### Gremlin1 detection in gastric cancer cell lines by western blot analysis

Protein detection of gremlin1 in gastric cancer cell lines was facilitated by western blot analysis. Gastric carcinoma cell lines MKN7, MHK45, MKN75, KATO-III and NUGC4 were purchased from the Japanese Physical and Chemical Institute, Tokyo, Japan. They were maintained in RPMI 1640 that was supplemented with 10% fetal bovine serum (FBS), 100 units/ml penicillin and 100 μg/ml streptomycin at 37 °C in a cell incubator. Cells were harvested by centrifugation, rinsed with phosphate buffered saline (PBS) and subjected to total protein extraction using an immunoprecipitation assay lysis buffer. Proteins were extracted from the cell lines for detection of gremlin1 by the western blot analysis. Equal number of cells was separated using SDS-polyacrylamide gel, then transferred to Hybond membrane and subsequently blocked overnight in 5% skimmed milk in TBS. For immunoblotting, the membrane was incubated overnight with rabbit antibody against gremlin1 (1:2000). It was then rinsed by TBST and incubated with anti-rabbit IgG conjugated to horseradish peroxidase for 15 min. Bands were visualized with X-ray film (Fuji, Japan) by ECL-Plus detection reagents. The membrane was subsequently washed with WB Stripping Solution (Nakarai, Tokyo, Japan) for 15 min and treated as described above except with anti-β-actin antibody (sc-47778, Santa Cruz, 1:4000) as the internal control.

### Evaluation of gremlin1 expression in gastric cancer

**Fig. 1:**
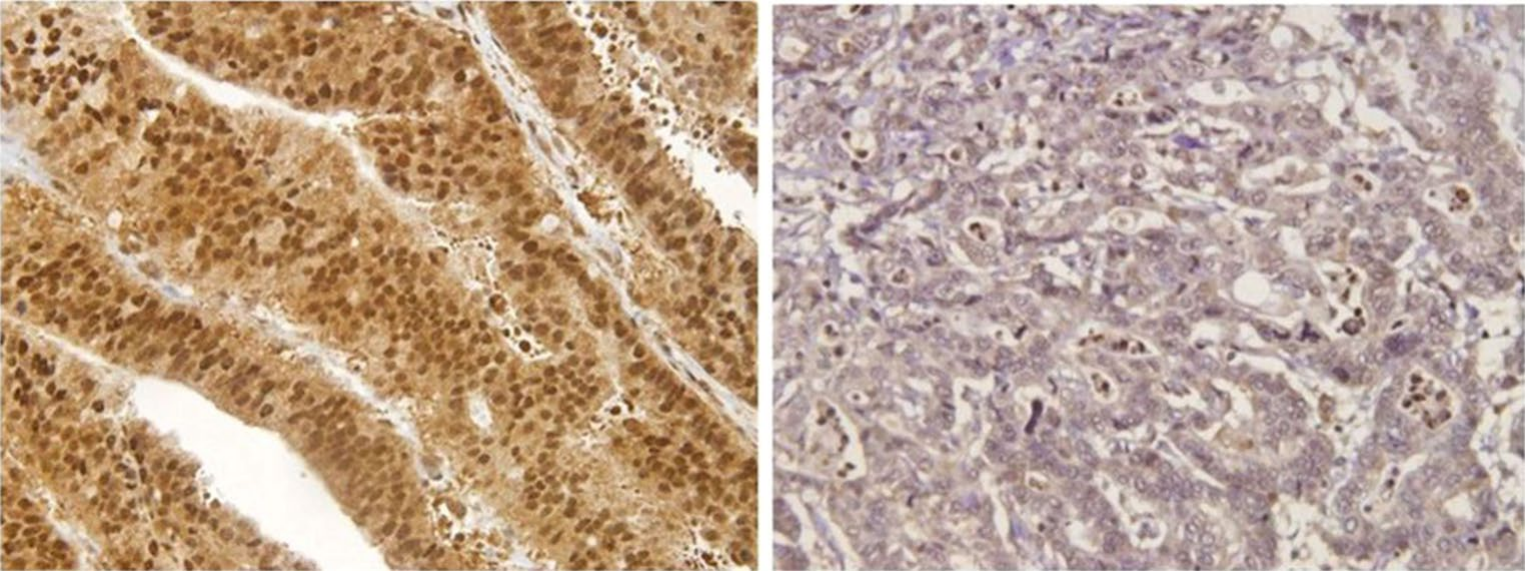
Gremlin1 expression in gastric cancer. Right: Gremlin1 expression was found in neither the cytoplasm nor nucleus in gastric cancer cells. We estimated as gremlin1-negative patients. Left: Immunohistochemically gremlin1 expression was identified in the cytoplasm and nucleus in gastric cancer cells. We estimated as gremlin1-positive patients

Gremlin1 expression was identified in the cytoplasm and nucleus of gastric cancer cells as well as stromal cells (Fig. 1). The quantification of gremlin1-positive cells was evaluated with high-power fields (HPFs). Briefly, gremlin1 positivity was calculated in ten representative HPFs in each tumor nest and at the invasive front of the tumor. All immunostained slides were evaluated by two independent observers (YY and SI), who were unaware of the clinical data and disease outcome. The gremlin1 expressions in gastric cancer were classified into four different staining categories: negative, weak, moderate and strong, according to a previous study [17]. We defined patients with negative and weak staining as the gremlin1-negative group and moderate and strong staining as the gremlin1-positive group. The correlation between clinicopathological factors and gremlin1 positivity was analyzed, which also included the patients’ survival rate.

### Statistical analysis

Statistical analysis between two groups was performed using the Chi-squared test. The Kaplan–Meier method was used for survival analysis, and the significant difference was evaluated by the log-rank test. Multivariate analysis was performed by the Cox proportional hazard model. *P* < 0.05 was considered statistically significant.

## Results

### Gremlin1 expression in gastric cancer tissues

Immunohistochemical gremlin1 expression was identified in the cytoplasm and nucleus in both the gastric cancer cell lines and surgical specimens (Fig. 1). We also identified the partial gremlin1 expression in adjacent normal gastric tissue by the immunohistochemical analysis. Immunohistochemical staining revealed gremlin1 positivity in both the nucleus and cytoplasm for all five cell lines. Based on the immunohistochemical evaluation described previously, 117 of 232 patients (50.4%) were classified as gremlin1 positive and the remaining 115 (49.6%) as gremlin1 negative.

### Gremlin1 protein expression in gastric carcinoma cell lines by western blot analysis

**Fig. 2:**
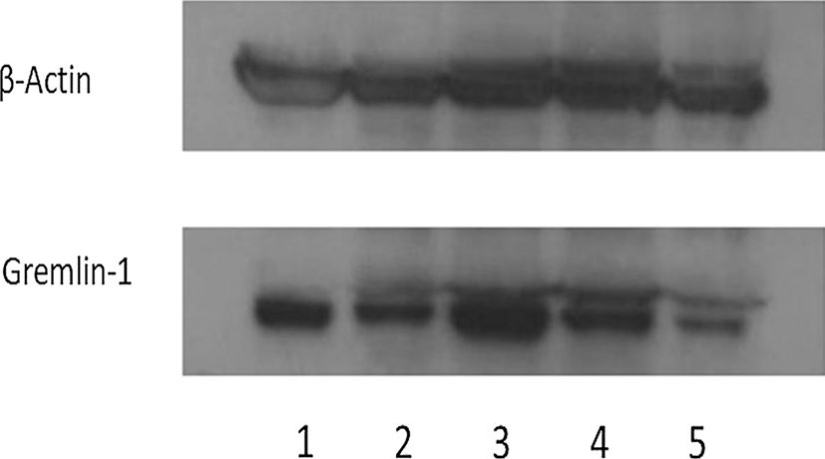
Gremlin1 detection in the five gastric cancer cell lines by western blot analysis. Gremlin1 protein was detected in the five gastric cancer cell lines by western blot analysis in various degree (1: MKN 7. 2: MKN45. 3: MKN74. 4: KATO-III. 5: FNUGC4)

All five cell lines had positive gremlin1 protein expression by western blot analysis (Fig. 2). Densitometric analysis revealed that the amount of gremlin1 protein was not correlated with tumor histology and metastatic ability (data not shown).

### Clinicopathological features of gastric cancer patients according to gremlin1

Gremlin1 expression negatively correlated with clinicopathological variables. Specifically, the gremlin1-negative group had significantly increased depth of tumor invasion, lymph node metastases and lymphatic and venous invasions than the gremlin1-positive group (*p* < 0.01). Moreover, the gremlin1-negative group had significantly more differentiated histology (*p* < 0.05) (Table 1). There was no significant difference between gremlin1 expression and other clinical parameters.

**Table 1:** Association between gremlin1 expression and clinical factors

Clinical factors	Expression of gremlin1		
	Positive	Negative	*p* value
	*n* = 117	*n* = 115	
Age			
< 65	44	40	0.656
≧ 65	73	75	
Gender			
Male	78	82	0.447
Female	39	33	
Tumor size (mm)			
< 50	55	34	< 0.01
≧ 50	62	81	
pT			
T1 (sm)/T2 (mp)	55	24	< 0.01
T3 (ss)/T4 (se)	62	91	
pN			
Yes	53	86	< 0.01
No	64	29	
Lymphatic invasion			
Yes	78	98	< 0.01
No	39	17	
Venous invasion			
Yes	51	81	< 0.01
No	66	34	
Histology			
Differentiated	50	68	< 0.05
Undifferentiated	67	47	

*pT* tumor depth of invasion, *pN* nodal involvement

### Prognostic impact of gremlin1 expression in gastric cancer

**Fig. 3:**
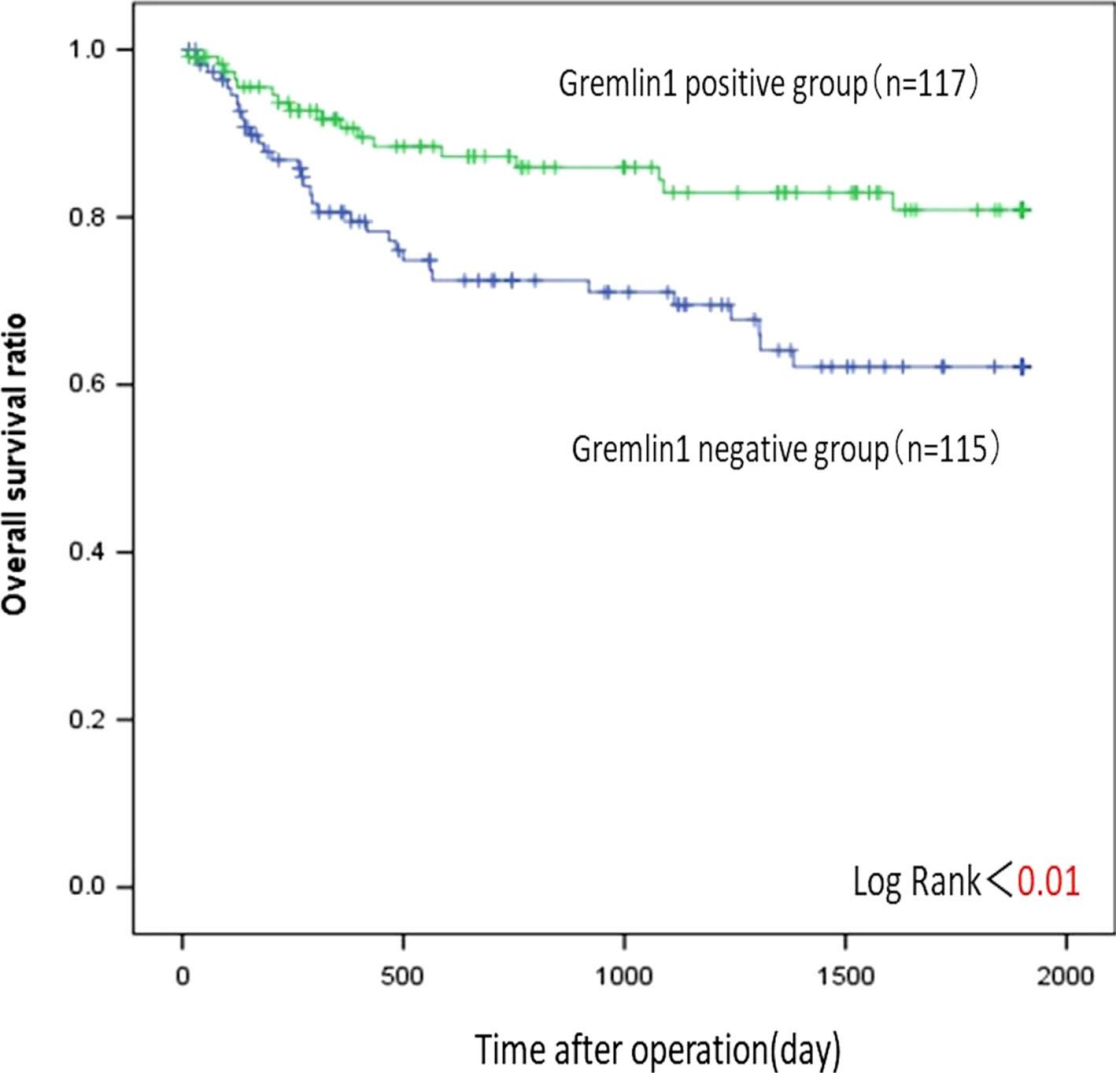
Survival curves of 232 gastric cancer patients according to gremlin1 expression. The patients with gremlin1-positive group showed significantly better postoperative outcome than those with gremlin1-negative group by univariate analysis (*p* < 0.01)

The gremlin1-positive group had better postoperative outcomes than the gremlin1-negative group (*p* < 0.01) (Fig. 3). The univariate analysis also revealed that postoperative survival was significantly affected by tumor histology, tumor depth, tumor size, lymph node metastasis and lymphatic and venous invasion (*p* < 0.01). The multivariate analysis showed that the depth of invasion, lymph node metastasis and tumor histology were independent prognostic markers; however, gremlin1 was not included (Table 2).

**Table 2:** Univariate and multivariate analysis of survival with clinical factors including gremlin1

Clinical factors	Univariate	Multivariate	Hazard ratio	95% confidence interval
Age	0.554	0.799	–	
Gender	0.953	0.594	–	
Histology	< 0.01	< 0.05	1.993	1.110–3.580
pT	< 0.01	< 0.05	4.584	1.315–15.977
Tumor size	< 0.01	0.28	–	
pN	< 0.01	< 0.01	3.68	1.370–9.885
Lymphatic invasion	< 0.01	0.395	–	
Venous invasion	< 0.01	0.136	–	
Gremlin1	< 0.01	0.076	–	

*pT* tumor depth of invasion, *pN* nodal involvement

## Discussion

In this study, we found gremlin1 positivity in the peritumoral gastric tissue by immunohistochemical analysis. Laurila reported that gremlin1 was also identified in the normal gastric gland [19]. Moreover, gremlin1 expression has been identified in not only normal tissue but in neoplastic cells, such as glioblastomas, hepatocellular carcinomas and diffuse large B-cell lymphomas [20]. Using immunohistochemistry, we identified gremlin1 expression in the cellular membranes and nuclei. The distribution pattern of gremlin1 in gastric cancer was similar to normal gastric epithelial cells; this may suggest an involvement with the normal function of the stomach. Their elevated expression in gastric cancer cells indicates the potential significance of the BMP-gremlin1 signaling. Gremlin1 may be involved in the carcinogenesis of gastric cancer, which is similar to the organogenesis of normal gastric epithelium.

Gremlin1 expression was negatively associated with aggressive parameters, such as tumor diameter, depth of tumor invasion, lymph node metastasis and vascular invasion. Vlodrop et al. showed that gremlin1 silencing in renal cell carcinoma led to aggressive tumor behavior [13], which is in agreement with our results. They investigated gremlin1 silencing by region iii methylation using RT-PCR and subsequently showed that the methylation of gremlin1 was associated with increased tumor size, stage, histological grade and decreased mean vessel density. Gremlin1 expression in gastric cancer cells may also act to inhibit proliferation and invasion of cancer cells through the BMP signaling pathway. In this study, we could not find evidence of CpG island methylation for gremlin1. Some gremlin1-positive patients may have had gremlin1 silencing. For future studies, analysis of gremlin1 m-RNA and methylation status should be performed to determine the clinical features of gremlin1-positive gastric cancer. Yui showed clinical features of positive gremlin1 expression in non-small cell lung cancer by quantitative real-time PCR and western blot [21], which was contradictory to our results. This may be due to organ specificity or difference in methodology.

As an antagonist of BMPs, gremlin1 has a role in regulating the development of the lung, limb, kidney and other normal organs [22, 23]. Langenfeld showed that BMP2 and BMP4 exert angiogenic activity, which may increase with the inactivation of the BMP-antagonist gremlin1 [24]. Recently, reports have indicated that gremlin1 has a BMP-independent role which is related to angiogenesis and tumorigenesis [25, 26].

In this report, we showed that gastric cancer with severe vascular invasion in the peritumoral area was significantly correlated with gremlin1-negative cancer. Furthermore, we showed that gremlin1 negativity contributed to poor clinical outcomes in gastric cancer. Two reports [13] showed the prognostic significance of gremlin1 expression by multivariate analysis. In this study, gremlin1 was not found to be an independent prognostic marker by multivariate analysis, which may have been due to the strong association of tumor depth of invasion and lymph node metastasis. Previously, we showed that the gremlin1 antagonist BMP7 in gastric cancer was one of the independent factors for poor prognosis [11]. Our data suggest that the prognostic impact of gremlin1 may alter the expression of BMP7 expression in consideration of the antagonist of gremlin1. More attention should be given to the synergistic effect of BMP7 expression with gremlin1-positive gastric cancer.

Karagiannis et al. demonstrated that CpG island methylation of gremlin1 can be detected in urine or serum samples, which can be used as a possible noninvasive marker [27]. Furthermore, serum gremlin levels can be used to indicate the biological behavior of tumors in gastric cancer. Biopsy specimens obtained from endoscopy may also inform the agonist and antagonist relation status between gremlin1 and BMP. In addition, Namkoong et al. showed the therapeutic value of the YWHAH protein which binds gremlin1 of gremlin1-positive carcinomas of the uterine cervix, lung, ovary, kidney, breast, colon and pancreas [28]. Gremlin1 may play an oncogenic role for these carcinomas. As of yet, we do not have a therapeutic strategy for gremlin1-positive gastric cancer patients based on our clinical data.

In conclusion, gremlin1 expression in gastric cancer directly reflects the biological behavior of the tumors. Gremlin1 expression may antagonize BMP7 signaling, and a combination of gremlin1 and BMP7 may contribute to a stronger prognostic marker in gastric cancer. Based on these results, gremlin1 may be used as a diagnostic or treatment tool for gastric cancer in the future.
